# Next generation genetics

**DOI:** 10.3389/fgene.2014.00322

**Published:** 2014-09-16

**Authors:** Mogens Fenger

**Affiliations:** Clinical Biochemistry, Molecular Biology, and Genetics, KBA339Hvidovre, Denmark

**Keywords:** epistasis, genetic networks, evolution, modeling, hidden structures

One of the goals in genetic research aims at identifying genes in biochemical and physiological processes to reveal genetic causes of rare and common diseases. Previous obstacles such as costly genotyping or sequencing have been reduced with the chip-based genomewide association studies (GWAS), now culminating with the latest toy—next generation sequencing methodologies (NGS). Concomitantly, computer technologies have evolved to an increasing use of multicore processors and distributed computing on large networks or grids. Although the technologies are not perfect, we now have unprecedented opportunities to perform genetic studies not possible just 10 years ago. The hype about these new technologies have been large, but all the promises have however not been fulfilled entirely as hoped for. Maybe because the hype has been more about the technologies as such, and less about their intended use. Millions of genetic variations have been detected by GWAS and NGS, but only a few have been linked to diseases—with almost no practical clinical significance. A major reason for this apparent deadlock is the inadequacy of the models used, which are based on the traditional “Mendelian” approach, in which one gene is supposed to have a main effect on a trait or a disease. However, most genes claimed to be associated with a disease have small effects and only a tiny fraction of the genetic variance has been captured.

In this short notice it is argued, why this traditional approach should be supplemented or even replaced by modeling approaches in accordance with the complexity of biological systems, if we shall have any reasonable hope to understand the genetics behind any trait and bring genetics into practical use in medicine for common diseases (Costanzo et al., 2010; Ramanan et al., 2012).

## Evolution, fitness, and epistasis

Evolution of phyla is a complex process governed by genomic as well as environmental factors (Marshall, 2006). Much theoretic and practical work about evolution are based on theories of adaptive landscapes of fitness and natural selection, as advocated by Fisher in his geometrical model of adaption (Fisher, 1930; Martin and Lenormand, 2006; Chevin et al., 2010; Weinreich and Knies, 2013). In this model fitness is determined in a multidimensional landscape of phenotypes or traits, on which a selection pressure is imposed that limits the number of viable phenotypes. Although the space of theoretically phenotypes increases with the complexity of an organism, this may come with a cost of decreasing adaptability (Fisher, 1930; Orr, 2000; Martin and Lenormand, 2006; Borenstein and Krakauer, 2008). The Fisher model(s) is not explicitly rooted in genetic models but rather considers the phenotypic pleiotropic effect of mutations, i.e., particular genes and loci are not formulated in the model. In contrast, Wrights formulation of evolution (Wright, 1920) can be described as a multidimensional mutational or genetic landscape in which each dimension corresponds to a specific locus. In “modern” terms these ideas can be formulated as the occurrence of stabilizing selection acting on the increasing mutational load possibly involving pleiotropic behavior of a given mutation, that is a mutation may affect several endophenotypes (Weinreich et al., 2006; Masel and Siegal, 2009). Pleiotropicity also means that a selection pressure imposed on one endophenotype may not only constrict the number of viable genotypes but also inflict a collateral selection on other endophenotypes and genes (Gavrilets and De Jong, 1993; Snitkin and Segre, 2011). The latter may be regarded as “innocent” bystanders and the preserved genotypes may just be those that happen to be around at the moment of selection.

Fitness is a measure of the capability of survival and reproduction of a species as the result of integrated action of many subprocesses conditional on the imposed selection pressure. However, less fitness may not necessarily result in an entire loss of a phenotype or trait, but may prevail and in fact increase fitness if the selection pressure changes. This scenario is supported by the long known fact that a mutation may have major effect in one genotypic background, but may only have a minor influence in another and hence escape purging by selection (Nijhout and Paulsen, 1997; Kouyos et al., 2006). The fitness landscape (or any other trait landscape) may thus be roughed with several local optima. This is clearly obvious in the landscape of species, but is also present within a species (Marshall, 2006).

For long the question has been if a mutation impose a pure additive effect on fitness or if epistasis (the effect of mutations in a gene or regulatory structure on the effects of other genes) is the prevalent driving force in evolution. The effect of any mutation (genic as well as exgenic including possible changes in epigenetic processes) may be increased, buffered, or ameliorated in particular genetic backgrounds, while having negative (even lethal) effect in other genetic backgrounds. Buffering is the essence in evolutionary theory of canalization and organismal robustness, in which the phenotype appears robust to mutational perturbations. Mutations may accumulate and appear as cryptic or neutral variations as long as they are not selected against (Masel and Siegal, 2009). These cryptic genetic variations may be revealed if some genetic or environmental changes happen affecting the fitness and then contribute to evolvability (McGuigan and Sgró, 2009).

Canalization (or buffering) implies that the phenotypic mean tends to be preserved when a mutation occurs, but the cost is diminished variation of the phenotype, as new (deleterious) mutations are buffered leaving less degrees of freedom of variation compared to the pre-mutational genotype. Thus, a particular phenotype representing a local maximum in the phenotypic landscape, is generated by an ensemble of genotypes, each depicting a path or trajectory of the genetic network. Generally, the probability of a given genetic path being accessible to generate a phenotype decreases with the number of mutations. However, as the number of paths increases exponentially with the number of mutations a large and increasing number of paths may eventually generate a phenotype. This hypothesis has been confirmed empirically (Dowell et al., 2010; Franke et al., 2011). These and many other studies have firmly established epistasis as a primary driving force in evolution and as a fundamental principle in governing biological processes (Rice, 1998; Segre et al., 2005; Weinreich et al., 2005, 2013; Bershtein et al., 2006; Borenstein and Krakauer, 2008; Pavlicev et al., 2008; Chevin et al., 2010; Lunzer et al., 2010; Breen et al., 2012; Huang et al., 2012; Hemani et al., 2013; Weinreich and Knies, 2013).

The mechanism behind interactions and epistasis has been extensively studied and includes concepts as sign epistasis (Weinreich et al., 2005), reciprocal epistasis (Poelwijk et al., 2011), and the expansion of the protein universe (Povolotskaya and Kondrashov, 2010) to mention just a few outstanding contributions. The reader is referred to the cited work and to the vast literature appearing now.

## The genetic and phenotypic spaces

Complex species like humans are organized in interacting and interdependent functional units called organs or multicellular tissues. This extends the complexity of the genetics to another level. Despite the constrains this impose, the phenotypic space is vast.

Suppose that a diploid organism like *Homo sapiens* with 23 sets of chromosomes only harbor one mutation in each chromosome. The theoretically number of gametes emerging by random segregation amounts to approximately 8.4 million. If all gametes are viable then the number of possible zygotes will be more than 7*10^13^ or more than 11.000 fold the number human beings ever lived on planet Earth. Most probably a vast amount of the gametes or zygotes are not viable, but nevertheless, genetic variations so far discovered runs in the millions. This maps to as many phenotypes and hence, two human beings will never be genetically identical.

Similarly, in a physiological process like blood pressure, which are regulated by say 100 interacting genes, more than 10^30^ networks with exactly the same topology can be constructed if just one mutation is present in each gene. This would map to as many physiological states and dynamics. Adding to the number of genes their alternative spliced forms, the vast number of posttranslational modifications of proteins, non-protein regulatory elements (metabolites, small regulatory RNAs), epigenetic modifications, non-genic regulatory and genome-organizing structures, and not the least interactions and communications between cells in a multicellular organisms like humans, the combinatorial space of interactions and hence phenotypes is (almost) infinite.

## Population structure and genetic networks

Two basic aspects must be addressed in population genetics: (1) biological processes even in its most simple forms are blue-printed in the genome of interacting networks of genes; (2) the expression of the biological processes and phenotypes are conditional on the genetic variations and their inherent epistasis.

**Figure 1:**
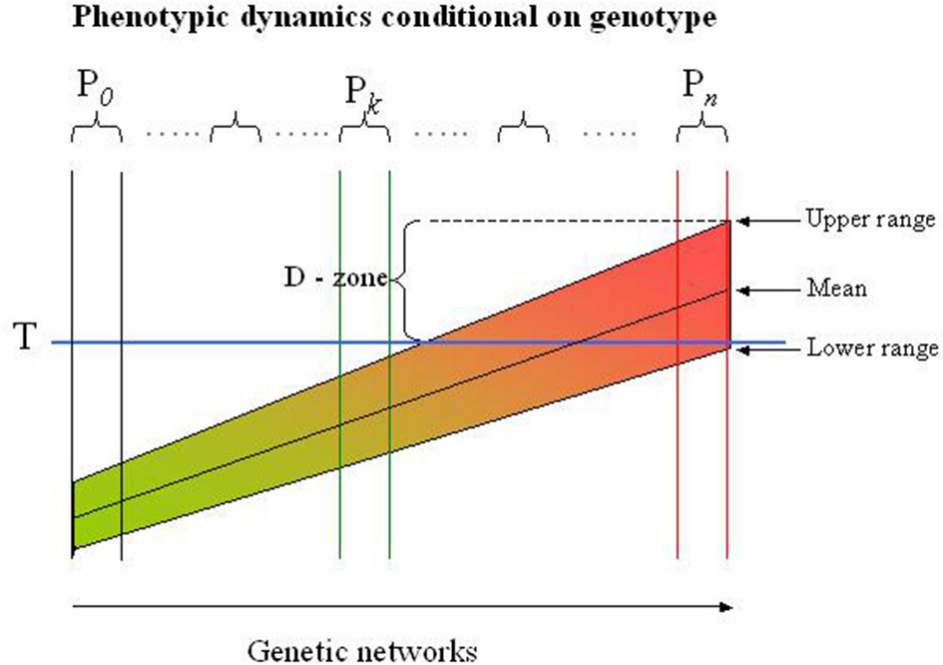
**Risk profile in genetic networks**. The population in this example is partitioned into more homogeneous subpopulations (P_*i*_) by the LCA-SEM procedure indicated by the sets of two vertical lines. Each subpopulation is genetically defined by an ensemble of networks with exactly the same topology but differs in genetic variations. The networks in each subpopulation arise by successive mutational incidences that are balanced (buffered) to generate a phenotype similar for all subjects in the subpopulation (see also the text). In reality the subpopulations represent different local maxima in a miltidimensional phenotypic landscape, but are for illustrative purposes collapsed to flat, two-dimensional presentation. The range of the phenotype (e.g., diastolic blood pressure) depends on extra-genetic factors, but can never exceed the range defined by the genotype. Thus, some subjects (genotypes) will never exceed the threshold (T), while others will experience the extreme phenotype (e.g., diastolic hypertension) regardless of extra-genetic factors. The D(anger) - zone indicates the subjects or subpopulations which may be classified in this examples as diastolic hypertensive. However, this may depend on the circumstances under which the blood pressure is measured, i.e., subjects may be classified as normotensive although they have a massive propensity to develop hypertension. Dichotomizing the trait in a population is thus a dubious affaire and compromise most association studies to the point that information of the genetics of, in this case diastolic blood pressure, is entirely lost.

The genetic networks coding a trait can be mapped as a “continuum” reflecting the physiological states they define (see the Figure 1). Neighboring networks can be distinguished by variations in one or several genes or non-genetic regulatory structures, but may appear physiological similar as most genetic variations have small effects. The sensitivity of a network to external factors is encoded in the genome, and it is the variations in the process-specific genes and regulatory structures that determines the range of the response to an external perturbation. Identifying genetic networks are neither simple, nor transparent: functional networks are multipartite structures and are not secluded entities but rather interconnected with other networks (e.g., the glucose and the fat metabolisms are highly intermingled processes). Nevertheless, it may be possible to define a reasonable number of sub-ensembles of networks to be interpretable.

## Heterogeneity

Population heterogeneity refers to the mixture of phenotypically homogeneous subpopulations, although in the extreme no subpopulation is truly homogenous as each subject harbors a unique genomewide genotype. A more or less well-defined phenotype thus comprise an ensemble of genotypes in the population. The initial task is then to cluster subjects into more physiological homogeneous subpopulations to increase the accuracy and power of the genetic analysis (Fenger et al., 2008, 2011). The application of appropriate cluster algorithms is generally ill posed, as no universal formal criteria for the best clustering is available. Many of the well-known classification procedures implement some data-reduction or feature selection (Saeys et al., 2007), but any manipulations of the data space are likely to result in loss of information and should be avoided.

Allocating subjects to subpopulations is an art of modeling hidden or latent variables as the number of subpopulations is not known *a priori*. A way to resolve this is by applying the concept of latent class (LCA) in a structural equation modeling framework (SEM) (Bollen, 1989; Muthen, 2002; Skrondal and Rabe-Hesketh, 2007; Fenger et al., 2008, 2011). The idea of the LCA-SEM approach is to model a physiological process, and therefore the most appropriate study population would be a random selection of subjects as each subject provide information of the physiological process. Genetic structures and variations are not necessarily modeled directly, but are embedded as latent variables in the SEM structure and are mapped or reflected by the measured variables. Modeling in this framework addresses two pivotal issues in complex data: resolving the heterogeneity in the population, and simultaneously evaluating the data structure within the sub-populations. This approach outperforms most other classification methods in almost all aspects (Magidson and Vermunt, 2002).

An emerging line of methods implement ensembles of classification functions (Polikar, 2006). These approaches are particularly attractive when features in multi-source or distributed data sets are partly or completely disjoint, or if access to data in data set is limited to a subset of objects. Thus, the problem of missing data and hence reduced power may be circumvented to some extent and represent a potential alternative to imputing missing genetic data.

Undoubtedly, new and promising methods will merge, in particular as theoretical ideas mentioned below are integrated in future developments.

## Inheritance: genes or information?

Understanding and integrating the wealth of genetic data in a biological and medical context requires new approaches and techniques. Fortunately many new approaches are emerging increasingly embracing the nature of biological systems. In particularly the recent developments in network theory (Dorogovtsev and Mendes, 2002; Newman, 2009) including concepts of modularity (Newman and Girvan, 2004), stochastic block modeling (Karrer and Newman, 2011), statistical mechanics (Reichardt and Bornholdt, 2006; Ronhovde and Nussinov, 2009), and information theory (Anand and Bianconi, 2010) are promising.

All these methods comprise passing of information that have its corollary in genetics. Stabilizing selection may give rise to prevailing linkage disequilibrium of genes within and across chromosomes (Fenger et al., 2011). Such linkage disequilibrium arise as a consequence of preserving physiological processes regardless of the physical structure of the genome. Thus, inheritance is not a simple matter of passing on genetic material, but rather to combine information harbored in the genome into a viable organism. Information theoretic approaches in genetics may therefore be more promising (how abstract it may be) than traditional association methods, although transformation of these theories to biological structures may not be always straightforward.

## Is validation in genetics actually possible?

Validation is a standard requirements in genetic association studies today. However, validation of an association of a genetic variant to a trait or disease is often not or only sporadically obtained and for that reason a gene may be dismissed as disease related (Ioannidis, 2007; Shriner and Vaughan, 2011), or even to be pivotal in a physiological process (Fenger et al., 2011; Spijkers et al., 2011). It should hopefully be clear from the discussions above that validation should be expected to be an exception. A non-validated association may simply indicate a local optimum in the phenotypic landscape that happens to be detected because the genotypes in a population are permissive for expressing an apparent main effect. It has indeed been demonstrated that the lack of validation actually suggest more complex genetic structures governs a trait (Greene et al., 2009; Fenger et al., 2011), and including epistasis in the analysis may eventual confirm the importance of non-validated associations.

In the end the importance of genetic variations should be confirmed by cellular experiments. If possible, studies should be done in the cells where the genes in a network has its effect (which we often do not know). A gargantuan endeavor and at the moment maybe wild-fetched, but eventually any genetic variation has to be substantiated in a real biological context - not just as a statistical phenomenon.

### Conflict of interest statement

The author declares that the research was conducted in the absence of any commercial or financial relationships that could be construed as a potential conflict of interest.
